# Effects of an isotonic beverage on autonomic regulation during and after exercise

**DOI:** 10.1186/1550-2783-10-2

**Published:** 2013-01-04

**Authors:** Isadora Lessa Moreno, Carlos Marcelo Pastre, Celso Ferreira, Luiz Carlos de Abreu, Vitor Engrácia Valenti, Luiz Carlos Marques Vanderlei

**Affiliations:** 1 UNIFESP - Federal University of São Paulo, Department of Medicine, Cardiology Division, São Paulo, SP, Brazil; 2 UNESP – State University Paulista, Department of Physical Therapy. Presidente Prudente, Paulista, SP, Brazil; 3 Laboratory of Scientific Writing, School of Medicine of ABC, Santo André, SP, Brazil; 4 Departamento de Medicina, Disciplina de Cardiologia, Universidade Federal de São Paulo (UNIFESP), Rua Napoleão de Barros, 715 Térreo, 04039-032, São Paulo, SP, Brazil

**Keywords:** Exercise, Rehydration solutions, Autonomic nervous system

## Abstract

**Background:**

With prolonged physical activity, it is important to maintain adequate fluid balance. The impact of consuming isotonic drinks during and after exercise on the autonomic regulation of cardiac function is unclear. This study aimed to analyze the effects of consuming an isotonic drink on heart rate variability (HRV) during and after prolonged exercise.

**Methods:**

Thirty-one young males (21.55 ± 1.89 yr) performed three different protocols (48 h interval between each stage): I) maximal exercise test to determine the load for the protocols; II) Control protocol (CP) and; III). Experimental protocol (EP). The protocols consisted of 10 min at rest with the subject in the supine position, 90 min of treadmill exercise (60% of VO_2_ peak) and 60 min of rest placed in the dorsal decubitus position. No rehydration beverage consumption was allowed during CP. During EP, however, the subjects were given an isotonic solution (Gatorade, Brazil) containing carbohydrate (30 g), sodium (225 mg), chloride (210 mg) and potassium (60 mg) per 500 ml of the drink. For analysis of HRV data, time and frequency domain indices were investigated. HRV was recorded at rest (5–10 min), during exercise (25–30 min, 55–60 min and 85–90 min) and post-exercise (5–10 min, 15–20 min, 25–30 min, 40–45 min and 55–60 min).

**Results:**

Regardless of hydration, alterations in the SNS and PSNS were observed, revealing an increase in the former and a decrease in the latter. Hydrating with isotonic solution during recovery induced significant changes in cardiac autonomic modulation, promoting faster recovery of linear HRV indices.

**Conclusion:**

Hydration with isotonic solution did not significantly influence HRV during exercise; however, after exercise it promoted faster recovery of linear indices.

## Background

Physical activity leads to increased metabolic rate and heat production [1], resulting in loss of water and electrolytes and glycogen depletion in the liver and muscles [1,2]. The loss of these elements may lead to dehydration, affecting physical performance and impairing health [3]. Fluid replacement using isotonic solution may attenuate or prevent many metabolic, cardiovascular, thermoregulatory and performance perturbations [4,5]. Moreover, according to Brouns et al., [6] and Coyle [7], sports drinks without caffeine can help to maintain physiological homeostasis.

Another aspect of risk related to exercise is failure of cardiovascular function, especially for practitioners who exercise infrequently [8]. It is known that reduced cardiac parasympathetic regulation associated with increased sympathetic activation may trigger malignant ventricular arrhythmias, and that systemic metabolic disorders (electrolyte imbalance, hypoxia), as well as hemodynamic or neurophysiological (fluctuations in the activity of the autonomic nervous system) disorders appear to play an important role in lethal arrhythmias [9]. In addition, the physiological overload imposed on the body is enhanced when exercise is associated with dehydration. According to Carter et al., [5], “the combination of these two factors suggests changes in the global cardiac autonomic stability”.

In combination with dehydration, exercise has been shown to cause post-exercise alterations in the baroreflex control of blood pressure [10]. Charkoudian et al., [10] demonstrated that even modest hypohydration (1.6% of body weight) can blunt baroreceptor control of blood pressure and that physiological responses were not observed following an intravenous infusion of saline to restore the plasma volume after exercise in the heat.

Although it is known that changes in the cardiovascular system are caused by hydration during and after exercise, few studies have evaluated the influence of hydration on the autonomic nervous system (ANS) and none have evaluated this influence when isotonic drink is also administered during and after prolonged exercise. Our purpose, therefore, was to evaluate the effects of hydration protocols on autonomic modulation of the heart in young people during and post-exercise. We hypothesized that hydration during exercise and recovery may attenuate autonomic changes induced by exercise and accelerate recovery. To test this hypothesis, we assessed linear indices of heart rate variability (HRV) in young men with and without isotonic solution intake (Gatorade, Brazil) containing carbohydrates (30 g), sodium (225 mg), chloride (210 mg) and potassium (60 mg) per 500 ml of the drink.

## Methods

### Subjects

Thirty-one healthy, young male volunteers (21.5 ± 1.8 yr) were investigated. All were active according to the International Physical Activity Questionnaire - IPAQ [11]. The study group excluded: smokers, individuals on medications that would influence cardiac autonomic activity; alcoholics, individuals with cardiovascular, metabolic and/or known endocrine disorders; and those with sedentary or insufficiently or overly active lifestyles, according to IPAQ criteria. No volunteers were excluded during the course of the experiment. Every individual signed a consent letter and was informed of the procedures and objectives of the study. The study’s procedures were all approved by the Research Ethics Committee of the Faculty of Science and Technology - FCT/UNESP (Number 168/2007).

### Experimental design

Subjects reported to the laboratory three days per week, at an interval of 48 h between visits. An incremental test was applied during the first visit, which was performed on a treadmill (Super ATL, Inbrasport, Brazil) according to the Bruce protocol [12]. To establish the baseline, volunteers were allowed to rest in a standing position on the mat before the test began. Once the test started, verbal encouragement was used in an attempt to obtain a maximum physical effort; the test was interrupted by voluntary exhaustion. To determine oxygen consumption (VO_2_), expired gases were analyzed using a regularly calibrated metabolic analyzer (VO*2000*, Medical Graphics, St. Paul, MN, USA) [13]. The VO_2_ peak was taken to be the highest VO_2_ achieved in the test. The HR reached at 60% of this value was used to determine the exercise intensity for the protocols, considering that gastric emptying is considerably disturbed at intensities above 70% of VO_2_ peak [14].

In subsequent visits, called control (CP) and experimental (EP) protocols, volunteers were allowed to rest in the supine position for 10 min, followed by 90 min of exercise (60% of VO_2_ peak) and 60 min of recovery. Volunteers were not given any fluids to drink during CP; however, they were given an isotonic solution (Gatorade, Brazil), containing carbohydrates (30 g), sodium (225 mg), chloride (210 mg) and potassium (60 mg) per 500 ml of the drink, to consume during EP. The isotonic solution was administered in 10 equal portions at regular intervals of 15 min from the fifteenth minute of exercise until the end of the recovery. The amount of isotonic solution administered during EP was based on the difference in body weight between before and after CP. This technique indicates that 1 g reduction in body weight is equal to 1 ml of fluid reduction [15].

For all visits, volunteers were instructed to avoid consuming caffeine 24 h before the procedures, to consume a light fruit-based meal 2 h before the tests, to have a good night’s sleep (7–8 h), to avoid strenuous physical exercise the day before the test and to be dressed in appropriate and comfortable clothing (shorts, shirt, shoes and socks) for physical exercise.

### Control and experimental protocols

The protocols were performed in a room under controlled temperature (26.0 ± 2.3°C) and humidity (55.1 ± 10.4%) between 3 p.m. and 6 p.m. to avoid circadian variation. To ensure the condition of initial hydration the volunteers drank water (500 ml) 2 h before both protocols [16]. The volunteers’ weight (digital scale Plenna, TIN 00139 MÁXIMA, Brazil) and height (stadiometer ES 2020 - Sanny, Brazil) were measured upon their arrival at the laboratory. The heart monitor was then strapped on each subject’s thorax over the distal third of the sternum. The HR receiver (Polar Electro - S810i, Kempele, Finland) was placed on the wrist for beat-to-beat HR measurements and for HRV analysis.

HR was analyzed at the following periods: final 10 min of rest; after 30, 60 and 90 min of exercise; after 5, 10, 20, 30, 40, 50 and 60 min of recovery.

The volunteers remained at rest in the supine position for 10 min and immediately their axillary temperature (thermometer BD Thermofácil, China) was measured. Subsequently, the subjects performed a treadmill exercise (60% of VO_2_ peak) for 90 min and were then allowed to rest in the supine position for 60 min for recovery. Axillary temperature was checked again immediately following exercise; the volunteers’ weight was measured again at the end of the recovery period.

Urine was collected and analyzed (10 Choiceline, Roche^®^, Brazil) at the end of EP and after measurement of final body weight. Urine density was used as a marker for hydration level [17].

### Heart rate variability indices analysis

HRV was recorded beat-to-beat through the monitoring process (Polar Electro - S810i, Kempele, Finland) at a sampling rate of 1000 Hz. During the period of higher signal stability, an interval of 5 min was selected, and series with more than 256 RR intervals were used for analysis, [18] following digital filtering complemented by manual filtering to eliminate premature ectopic beats and artifacts. Only series with more than 95% sinus rhythm were included in the study [19]. To analyze HRV in the frequency domain, we used the low (LF) and high frequencies (HF) spectral components in normalized units (nu) and ms^2^, and the LF/HF ratio, which represents the relative value of each spectral component in relation to the total power, minus the very low frequency (VLF) components [18]. Normalizing data of the spectral analysis can be used to minimize the effects of changes in the VLF band. This is determined by dividing the power of a given component (LF or HF) by the total power spectrum, minus the VLF component and multiplied by 100 [18].

We considered the following range: LF: 0.04 – 0.15 Hz and; HR: 0.15 – 0.4 Hz. The spectral analysis was calculated using the Fast Fourier Transform algorithm [20]. Analysis in the time domain was performed by means of SDNN (ms) [standard deviation of normal-to-normal RR intervals] and RMSSD (ms) [root-mean square of differences between adjacent normal RR intervals in a time interval] [18].

HRV indices were analyzed at the following moments: M1 (final 5 min rest), M2 (25 to 30 min after exercise), M3 (55 to 60 min after exercise), M4 (85 to 90 min after exercise), M5 (5 to 10 min of recovery), M6 (15 to 20 min recovery), M7 (25 to 30 min recovery), M8 (40 to 45 min recovery) and M9 (55 to 60 min recovery). Series with more than 256 RR intervals were used for analysis (Task Force, 1996). We used Kubios HRV version 2.0 software to analyze these indices [21].

### Statistical analysis

Gaussian distribution of the data was verified using the Shapiro-Wilks test. For comparisons between protocols (Control vs. Experimental) and moments (M1, M2, M3 and M4 during exercise and M1 vs. M5, M6, M7, M8, M9 during recovery) two-way repeated measures analysis of variance was applied, followed by the Bonferroni post-test for parametric distributions or Dunn’s post-test for non-parametric data. The repeated-measures data were checked for sphericity violation using Mauchly’s test and the Greenhouse-Geisser correction was conducted when sphericity was violated. Significance level was set at p < 0.05 for all tests. SPSS (version 13.0) software (SPSS Inc., Chicago, IL, USA) was used for statistical analysis. The calculation of the power of the study based on the number of subjects analyzed and a significance level of 5% (two-tailed test), guaranteed a test power higher than 80% to detect differences between the variables.

## Results

The anthropometric characteristics of the subjects and their responses obtained during the incremental test are described in Table 1, while Table 2 shows data regarding body mass and temperature in CP and EP. We observed weight loss and increased body temperature in CP (Table 2). The percentage of body weight loss in CP was 2.0 ± 0.6%, while in EP it was −0.2 ± 0.7%. The average consumption of isotonic solution was 1.4 ± 0.5 L in EP. The density of urine (1.018 ± 0.004) evaluated at the end of EP confirms that the volume of solution intake was sufficient to maintain the subjects at euhydrated status [17].

**Table 1:** Subject characteristics

**Variables**	**Mean ± Standard deviation**	**Minimum/Maximum**
**Anthropometric data**		
Age (yr)	21.5 ± 1.8	[18-25]
Body mass (kg)	72.6 ± 11.5	[53.8 – 95.3]
Height (m)	1.7 ± 0.1	[1.6 – 1.9]
BMI (kg/m^2^)	23 ± 2.8	[16.8 – 28.1]
**Incremental test**		
VO_2peak_ (L.min^-1^)	3.3 ± 0.6	[2.0 – 5.1]
60%VO_2peak_ (L.min^-1^)	2.0 ± 0.3	[1.2 – 3.0]
HR (bpm)	160.7 ± 10.7	[139–179]

**Legend:** BMI = body mass index; VO_2peak_ = peak oxygen consumption; HR = heart rate; bpm = beats per minute.

**Table 2:** Values of body mass and temperature in control and experimental protocols

**Variable**	**Time**	**Control protocol**	**Experimental protocol**
		**mean ± standard deviation [minimum – maximum]**	
**Body mass (kg)**	**Before the protocol**	73.0 ± 11.5 [54.7 – 96.1]	72.9 ± 11.5 [53.5 – 96.6]
	**After the protocol**	71.5 ± 11.3 [53.6 – 94.2]	73.0 ± 11.5 [53.5 – 97]
**Body temperature (°C)**	**Before exercise**	36.4 ± 0.4 [35-38]	36.3 ± 0.3 [35 – 36.9]
	**After exercise**	37.2 ± 0.5 [35.5 – 38]	36.8 ± 0.4 [36-38]

**Figure 1:**
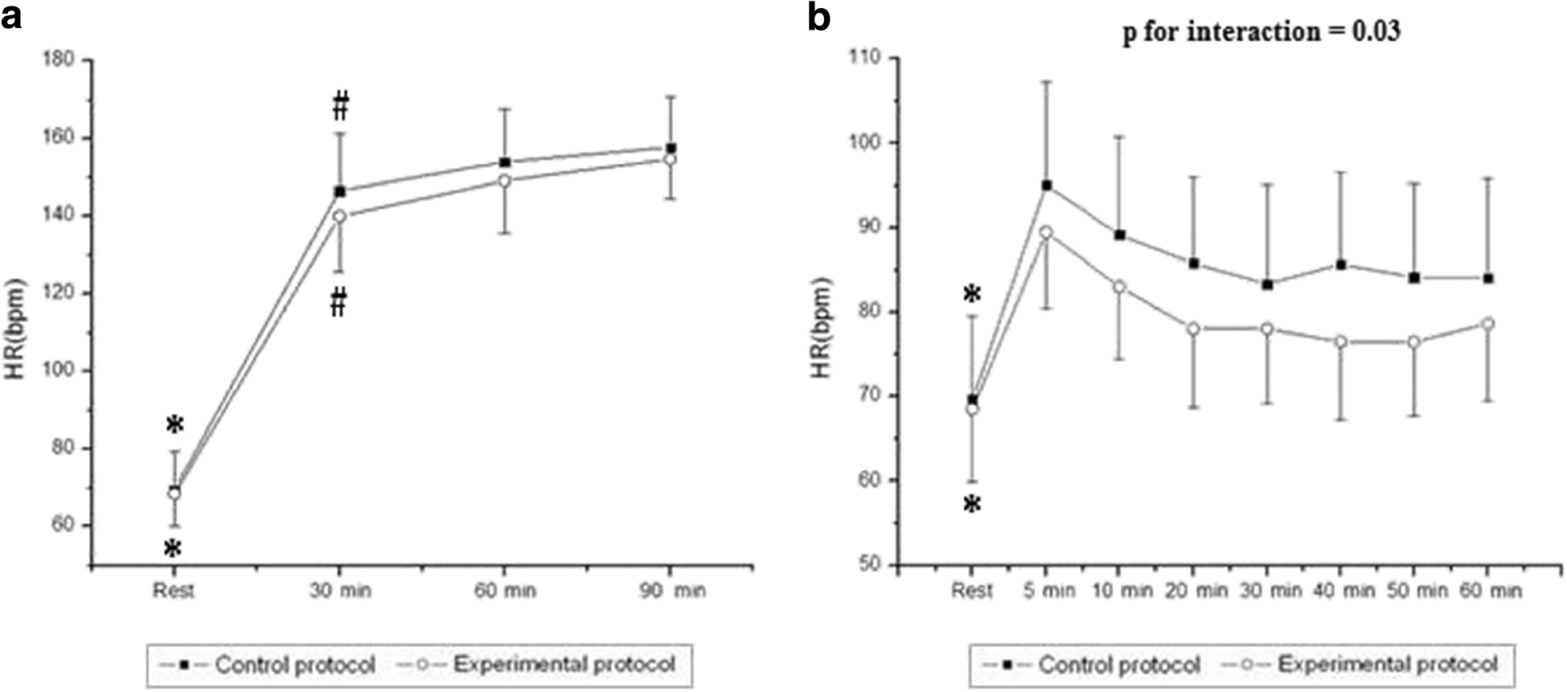
**Values are means ± standard deviation.** Heart rate (HR) during exercise (**a**) and recovery (**b**) and the comparison in control and experimental protocols; *Different from all the times of exercise and recovery (p<0.05); #Different from 90 min (p<0.05).

Figure 1 shows HR values during exercise and recovery. During exercise, we observed the effect of time (p < 0.001) on HR, however, there was no effect among protocols (p = 0.10). There was no interaction between time and protocol (p = 0.34). We noted that HR was significantly increased at 30, 60 and 90 min of exercise compared to rest, and significantly decreased at 30 min compared to 90 min in both CP and EP. In the recovery period, we observed the effects of time (p < 0.001), protocol (p = 0.008) and time and protocol interaction (p = 0.03) on HR, which suggests better recovery in the hydrated protocol. In both protocols, we noted that HR was significantly lower at rest, when compared to each minute of recovery, and after 60 min of recovery HR did not return to baseline.

**Figure 2:**
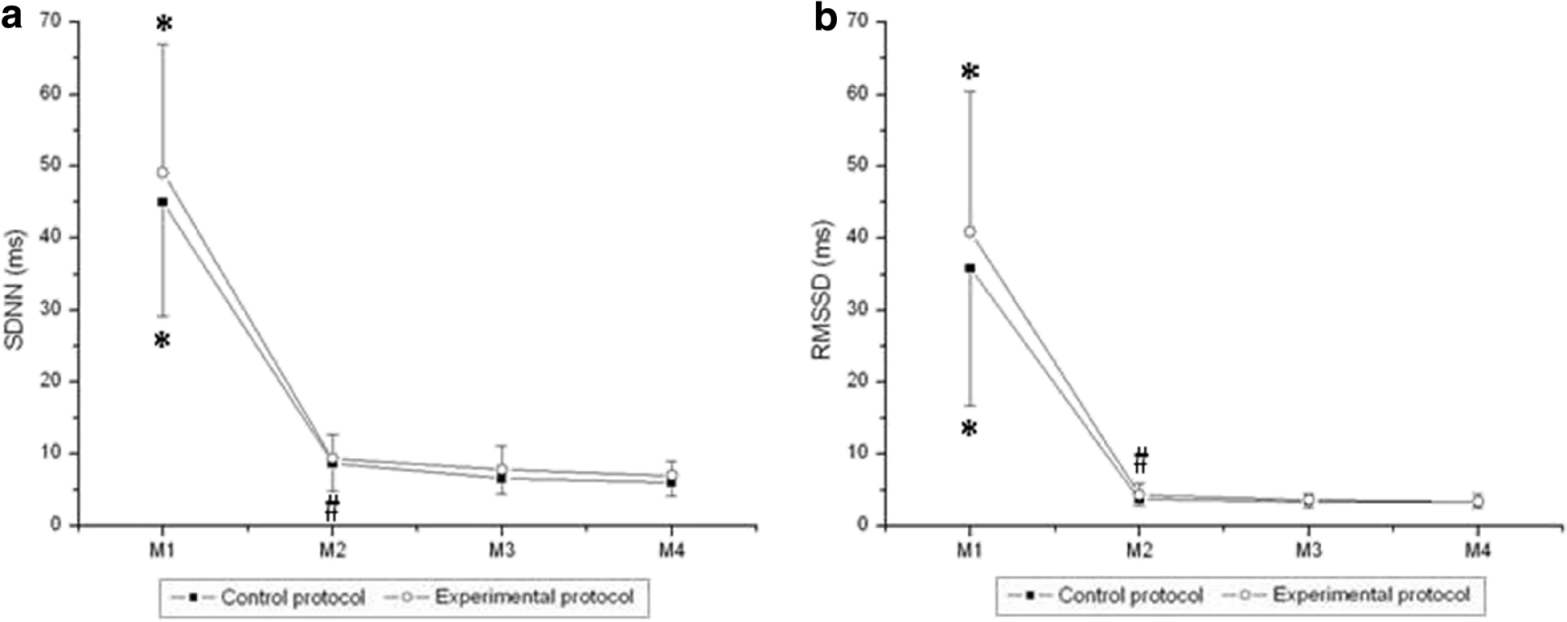
**Values are means ± standard deviation.** SDNN (**a**) and RMSSD (**b**) during exercise and the comparison in control and experimental protocols. Final 5 minutes of rest (M1) and minutes of exercise: 25^th^ to 30^th^ (M2), 55^th^ to 60^th^ (M3), 85^th^ to 90^th^ (M4). *Different from M2, M3 and M4 (p<0.05). #Different from M4 (p<0.05).

**Figure 3:**
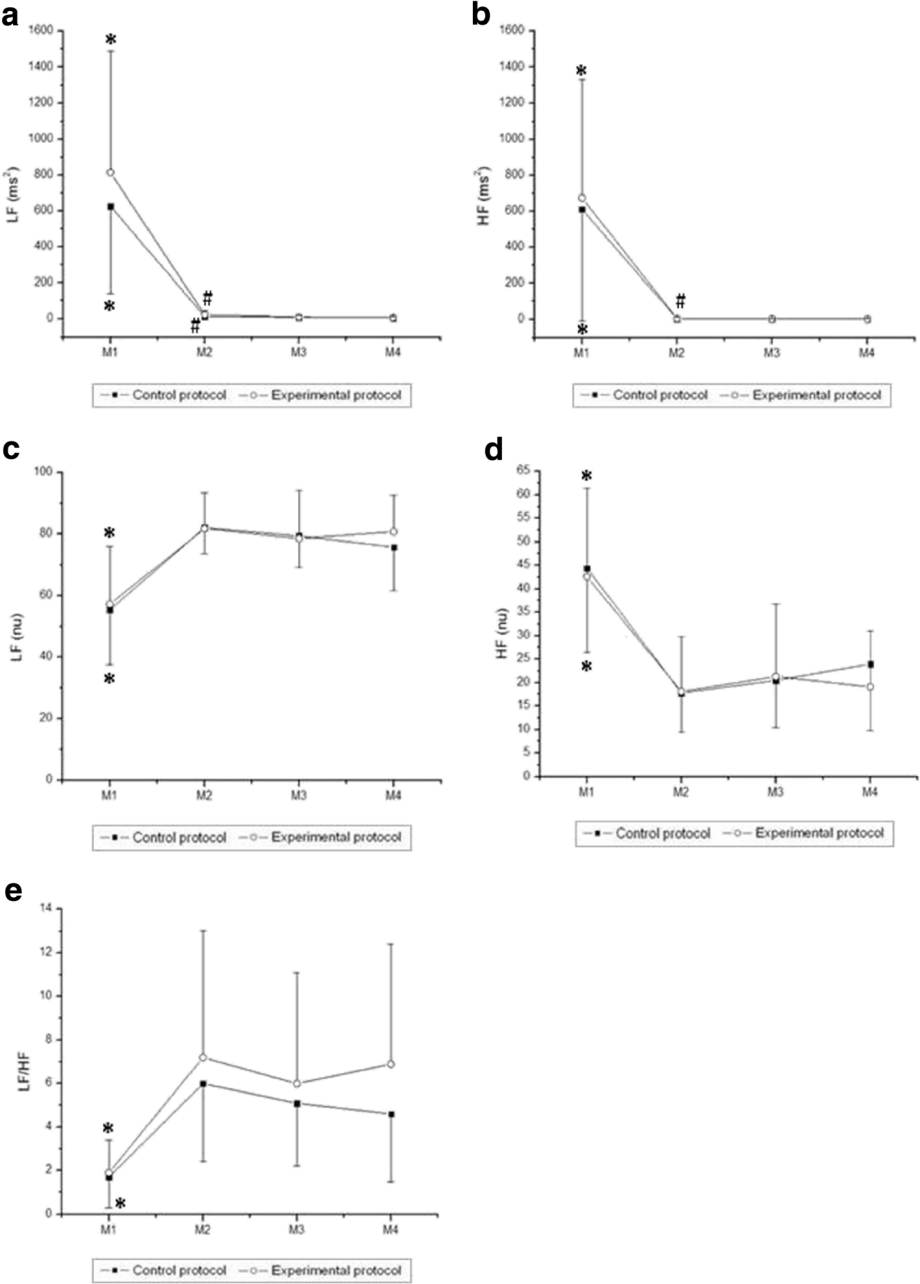
**Values are means ± standard deviation.** LFms^2^ (**a**), HFms^2^ (**b**), LFnu (**c**), HFnu (**d**) and LF/HF (**e**) during exercise and the comparison in control and experimental protocols. Final 5 minutes of rest (M1) and minutes of exercise: 25^th^ to 30^th^ (M2), 55^th^ to 60^th^ (M3), 85^th^ to 90^th^ (M4). *Different from M2, M3 and M4 (p<0.05). # Different from M4 (p<0.05).

Figures 2 and 3 show the behavior of HRV indices in time and frequency domains, respectively, during exercise. There was a moment effect for the time domain indices (SDNN and RMSSD; p < 0.001). No effects were observed between the protocols (SDNN, p = 0.12; RMSSD, p = 0.24) and in the time and protocol interaction (SDNN, p = 0.49; RMSSD, p = 0.32). We noted that SDNN (ms) and RMSSD (ms) were significantly decreased at M2, M3 and M4 of exercise in both CP and EP compared to M1 (rest). In addition, there was a decrease in the SDNN (ms) for CP and the RMSSD (ms) in EP at M2 of exercise compared to M4 of exercise.

Likewise, we observed a moment effect in all indices in the frequency domain (p < 0.001). No effects were observed for those indices between the protocols [LF (ms^2^), p = 0.18; HF (ms^2^), p = 0.69; LF (nu), p = 0.47; HF (nu), p = 0.47], except for the LF/HF ratio (p = 0.04). There were no interactions between time and protocol [LF (ms^2^), p = 0.22; HF (ms^2^), p = 0.70, LF (nu), p = 0.56; HF (nu), p = 0.56, LF/HF, p = 0.47]. Regarding the comparison between moments, we observed that LF (ms^2^), HF (ms^2^) and HF (nu) were significantly higher at M1 (rest) compared to M2, M3 and M4 of exercise in both CP and EP. LF (nu) and LF/HF were significantly lower at M1 compared to M2, M3 and M4 of exercise in both CP and EP. Moreover, LF (ms^2^) was significantly higher at M2 of exercise compared to M4 of exercise in both CP and EP, while HF (ms^2^) was significantly higher at M2 of exercise compared to M4 of exercise in EP.

**Figure 4:**
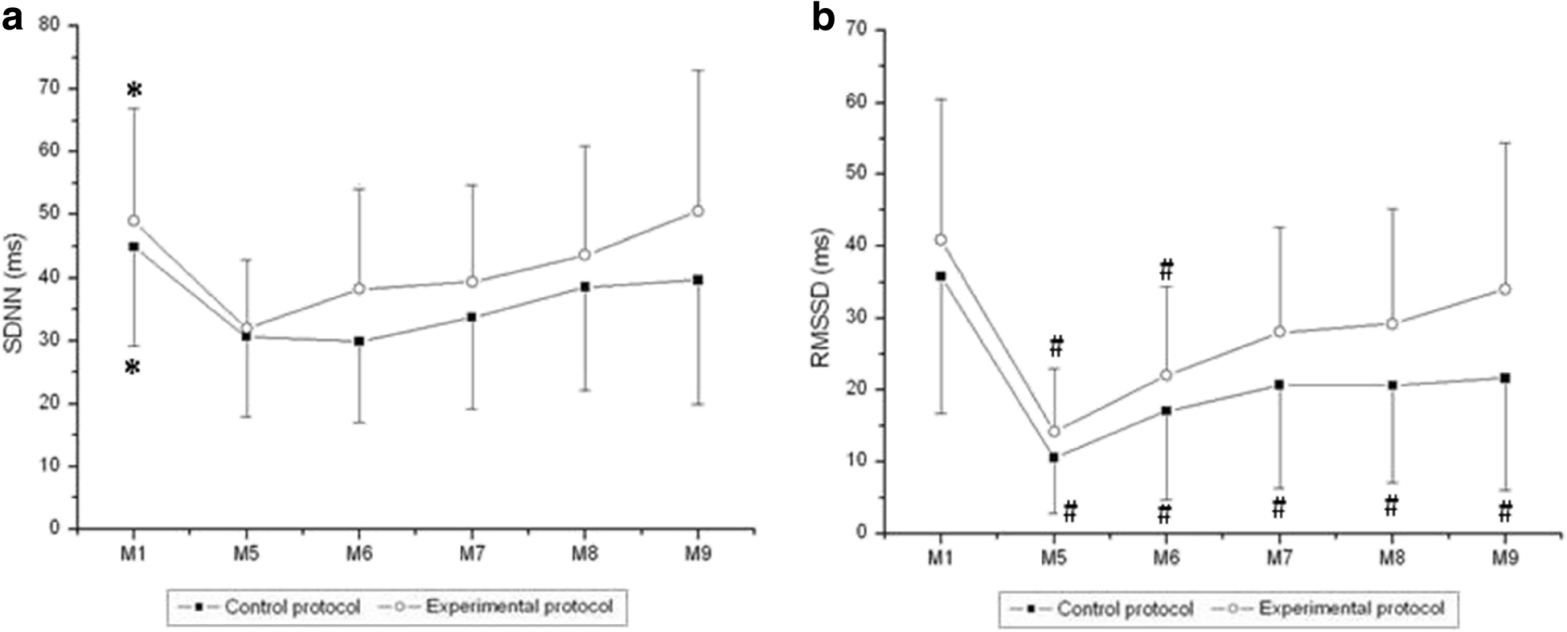
**Values are means ± standard deviation.** SDNN (**a**) and RMSSD (**b**) during recovery and the comparison in control and experimental protocols. Final 5 minutes of rest (M1) and minutes of recovery: 5^th^ to 10^th^ (M5), 15^th^ to 20^th^ (M6), 25^th^ to 30^th^ (M7), 40^th^ to 45^th^ (M8), 55^th^ to 60^th^ (M9). *Different from M5, M6, M7, M8 and M9 (p<0.05). #Different from M1 (p<0.05).

**Figure 5:**
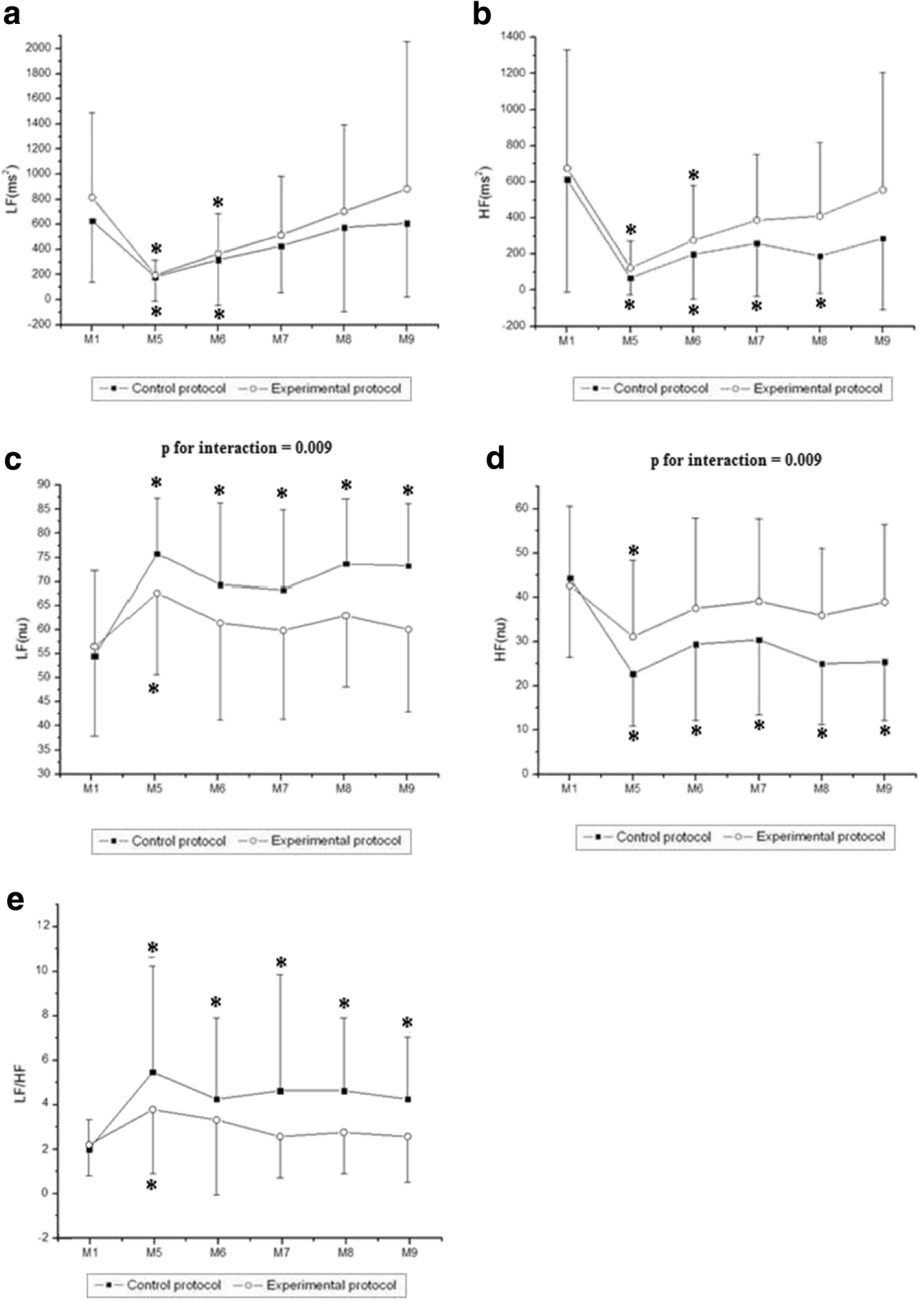
**Values are means ± standard deviation.** LFms^2^ (**a**), HFms^2^ (**b**), LFnu (**c**), HFnu (**d**) and LF/HF (**e**) during recovery and the comparison in control and experimental protocols. Final 5 minutes of rest (M1) and minutes of recovery: 5^th^ to 10^th^ (M5), 15^th^ to 20^th^ (M6), 25^th^ to 30^th^ (M7), 40^th^ to 45^th^ (M8), 55^th^ to 60^th^ (M9). *Different from M1 (p<0.05).

Figures 4 and 5 present the behavior of the HRV index in the time and frequency domains, respectively, during recovery. In relation to the time domain indices, we observed moment effects in the analyzed indices (SDNN and RMSSD, p < 0.001). Regarding the comparison of the SDNN index between recovery and rest (ms), it was significantly reduced at M5, M6 and M7 of recovery compared to M1 (rest) in both CP and EP. Regarding RMSSD (ms), it was significantly reduced at M5 and M6 of recovery compared to M1 (rest) in EP whereas it was significantly decreased at M5, M6, M7, M8 and M9 of recovery compared to M1 (rest) in CP. The effect of the protocol on RMSSD (ms) (p = 0.03) was also observed and no time and protocol interaction.

In relation to the frequency domain, time effect was observed in all indices analyzed (p < 0.001) and also the effect of the protocol on HF (nu) (p = 0.02), LF (nu) (p = 0.02) indices and LF/HF (p = 0.01) ratio. Interactions between time and protocol were observed in the LF and HF indices in normalized units (p = 0.009), suggesting better recovery in the hydrated protocol, as shown in Figures 5c and 5d. The LF (ms^2^) index was reduced at M5 and M6 of recovery compared to M1 (rest) in both CP and EP. HF (ms^2^) was significantly reduced at M5, M6, M7 and M8 of recovery compared to M1 (rest) in CP, while it was significantly decreased at M5 and M6 of recovery compared to M1 (rest) in EP. In relation to LF (nu), it was significantly increased at M5, M6, M7, M8 and M9 of recovery compared to M1 (rest) in CP, whereas it was significantly increased at M5 of recovery compared to M1 (rest) in EP. HF (nu) was significantly reduced at M5, M6, M7, M8 and M9 of recovery compared to M1 (rest) in CP while it was significantly lower at M5 of recovery compared to M1 (rest) in EP. LF/HF ratio was significantly higher at M5, M6, M7, M8 and M9 of recovery compared to M1 (rest) in CP and significantly increased at M5 of recovery compared to M1 (rest) in EP.

## Discussion

The results obtained in the present study demonstrated that the hydration protocol, despite producing lower alterations in the HRV indices, was insufficient to significantly influence HRV indices during physical exercise. However, during the recovery period it induced significant changes in the cardiac autonomic modulation, promoting faster recovery of HRV indices.

During exercise, the analysis of RMSSD (ms) and HF (nu), which predominantly reflects the parasympathetic tone of the ANS [22], showed higher but not significantly increased values when isotonic solution was administered. Studies indicate that factors linked to decreased vagal modulation in dehydrated individuals include attenuation of baroreceptor responses, difficulty in maintaining blood pressure and elevated levels of plasma catecholamines during exercise [10,23,24]. We expected that these factors may have influenced the lower values of RMSSD (ms) and HF (nu) in CP. Additionally, during exercise SNS activity predominated over vagal activity in both CP and EP. This mechanism occurs to compensate the body’s demands when exposed to exercise [25]. The increase in HR due to increased metabolism is associated with reduced global HRV [26], which was also observed in our study.

The SDNN index (ms), which reflects global variability, i.e., both vagal and sympathetic modulation [22], was reduced during exercise. The isotonic solution intake produced a smaller, though statistically insignificant, reduction in this index. It is possible that factors leading to the reduction of vagal modulation in dehydrated individuals [10,23,24] influenced the SDNN (ms) responses. Reduction in global HRV is expected during exercise [27], since it increases heart rate, stroke volume, cardiac output and systolic blood pressure, in order to supply the metabolic requirements. This mechanism may explain the LF (nu) increase during exercise, an index that is predominantly modulated by the sympathetic activity [22], and also the LF/HF ratio increase, which expresses the sympathovagal balance [22]. According to Mendonca et al., [28], the increase in the spectral indices suggests sympathetic activation during exercise at low and moderate intensities. Javorka et al., [29] reported similar findings - they investigated the HRV of 17 individuals subjected to 8 min of the step test at 70% maximal potency, and reported reduced SDNN (ms), RMSSD (ms) and HF and increased LF during exercise.

During exercise, as a consequence of reduced cardiac vagal activity, the reduction of global HRV is accompanied by a decrease in absolute power (ms^2^) of the spectral components [26]. This behavior was also observed in the present study: LF (ms^2^) and HF (ms^2^) indices decreased during exercise compared to rest, regardless of the administration of isotonic solution. The literature indicates that both spectral indices decreased exponentially according to exercise intensity [30]. Therefore, we expected minimal changes to be observed in these indices due to the work load maintenance during exercise in our study.

Similar results for SDNN (ms) and RMSSD (ms) were observed by Casties et al. [31], when 7 young individuals performed 3 consecutive 8 min stages at 40%, 70% and 90% of VO_2_ peak. However, contrary to our findings, they showed reduced levels of LF (nu) and LF/HF and an increase in HF (nu) at all intensities. The authors believe that it was due to the mechanical effect of hyperventilation on the sinus node, as well as synchronization between heartbeats, breathing and cycling. It is possible that different types of physical exercises (intensity and duration) contributed to these conflicting results. Additionally, since the HRV was extremely low during exercise and the LF/HF ratio is calculated using the ratio of two very small values, the data obtained from this relationship may be uncertain or highly sensitive to changes in the LF and HF indices, which may account for the conflicting results.

Although not significant, HR was higher when no fluid was ingested during exercise. Hamilton et al. [32] showed an increase in HR (10%), and reduced stroke volume (15%) when subjects performed 2 h of exercise without any fluid intake. When Gatorade powder fluid was administered, HR increased to 5% and stroke volume remained unchanged. This behavior observed in our study may be related to the “cardiovascular drift” phenomenon. Cardiovascular drift is characterized by findings of decreasing stroke volume and mean arterial pressure, rising heart rate, and stable cardiac output during sustained constant-load exercise [33,34]. A study in adults indicated that when dehydration is prevented by fluid intake, this pattern is altered, with no change in stroke volume and a progressive rise in cardiac output [33].

When analyzed during the recovery period, the indices that reflect the predominance of vagal activity, RMSSD (ms), HF (ms^2^) and HF (nu) presented a gradual increase and rapid recovery in approximately 25 min when the individuals were hydrated. Conversely, there was no complete recovery of these indices when the individuals were not hydrated. In addition, LF (ms^2^) and LF (nu), which predominantly reflect sympathetic nerve activity, also recovered faster in EP, especially LF (nu), which returned to baseline levels 15 min post-exercise. In CP, although LF (ms^2^) behavior was similar to that observed in EP, LF (nu) did not recover, suggesting sympathetic predominance in unhydrated subjects. Additionally, there was significant interaction between moments and protocols for the LF (nu) and HF (nu) indices, suggesting better post-exercise recovery in the experimental protocol.

The maintenance of volume and plasma osmolality associated with conservation of body temperature possibly influenced the recovery of HRV indices, which were evaluated in both time and frequency domains when isotonic solution was administered continuously after exercise. On the other hand, plasma hyperosmolality and increased body temperature, factors associated with hypohydration, possibly hampered the recovery of autonomic variables to baseline in CP. Hypohydration occurs during conditions of reduced intravascular volume and plasma hyperosmolarity, which trigger increased sympathetic activity and baroreflex control in order to protect against hypotension [35]. Charkoudian et al. [10] also observed that the combination of exercise and dehydration caused tachycardia and orthostatic intolerance after exercise in healthy subjects.

Changes in plasma osmolality are expected to influence baroreflex control of sympathetic nerve activity. Wenner et al., [36], after isolating the effect of increased plasma osmolality on baroreflex control, noted that when the intravascular volume was maintained, administration of hypertonic saline (3% NaCl) increased baroreflex control of sympathetic activity in humans compared to isotonic saline solution (0.9% NaCl). Scrogin et al., [37] also demonstrated that a 1% fall in plasma osmolality resulted in a 5% decrease in sympathetic outflow. Additionally, heat stress, which is increased by exercise and hypohydration, was associated with decreased cardiac vagal modulation [24]. Finally, Crandall et al., [24] also reported that reduced parasympathetic activity and increased sympathetic activity probably contribute to the rise in HR due to hyperthermia.

According to our results, the LF/HF ratio confirms the sympathetic predominance in unhydrated subjects in the recovery period. The sympathovagal balance was lower in EP compared to CP at 15 min, indicating the recovery of this index in the hydrated condition. Yun et al., [38] reported that hydration can reduce the sympathovagal ratio by reducing sympathetic activity through modulation of baroreceptors.

The influence of hypohydration and the combined effect of hydration status and exercise performance in the heat on the ANS were also studied by Carter et al., [5]. Five euhydrated and dehydrated subjects (4% loss of body weight) were studied at rest (sitting for 45 min), during exercise (90 min on a cycle ergometer at 60% of VO_2_ peak) and recovery (45 min post-exercise rest). Hypohydration reduced LF, VLF and LF/HF ratio, while HF was higher. Despite the fact that this condition positively influenced the vagal component (HF), the global reduction of HRV and attenuation in LF and HF oscillations observed post-exercise suggest a deleterious effect of dehydration on autonomic cardiac stability.

The continuous ingestion of isotonic solution, post-exercise, improved HR recovery. There was significant interaction between moments and protocols for the HR, suggesting better post-exercise recovery in the experimental protocol. It is suggested that hydration decreases sympathetic activity, which probably arises from a secondary effect of vagal afferent activity increased in response to modulation of baroreceptors during gastric distension [10,38]. This aspect may have influenced the pattern of HR response observed in this study when isotonic solution was ingested.

In the present study, no hydration also reduced global HRV after exercise. In relation to the SDNN (ms), despite presenting similar behavior in both conditions, higher values were displayed in the hydrated condition. This finding confirms the influence of hydration on post-exercise cardiac autonomic stability.

This study has some limitations. The minimum interval between the execution of control and experimental protocols was adhered to, however, some collections were completed over a period longer than a week, which may hinder the interpretation of the variables studied. Urine density was not determined at the end of the control protocol in this study, even though this might have contributed to the consolidation and interpretation of results. However, we were unable to collect urine from the subjects, as they were unable to urinate because they were not hydrated. Another important aspect refers to the use of supine rest and recovery conditions, considering that this exercise was performed in the upright position. Although we chose to compare rest and exercise in different positions, we believed that the modifications produced in the parameters during exercise were not influenced by the postural change. However, in addition to being more tolerable for the volunteer, the choice of the supine position during the recovery period has not impaired the results since the parameters were compared to a baseline, with subjects in the same position.

Considering the importance of the issue presented, other studies are in progress to evaluate the influence of water intake on cardiac autonomic modulation and cardiorespiratory parameters. Water ingestion provides rapid gastric emptying, requires no adaptation to the palatability of the solution and offers an economic alternative [39], aspects that are important in the context of hydration during and after exercise. These studies will allow us to evaluate the influence of water intake as a rehydration drink and to compare the effects of the ingestion of isotonic solutions and water as a means of rehydration on cardiac autonomic modulation. Such studies may enrich the knowledge in exercise physiology.

## Conclusions

We concluded that regardless of hydration status, the exercise protocol caused alterations in cardiac autonomic modulation, characterized by increased sympathetic and decreased parasympathetic activity. Although the isotonic solution administered (Gatorade, Brazil), containing carbohydrates (30 g), sodium (225 mg), chloride (210 mg) and potassium (60 mg) per 500 ml of the drink, generally produced lower alterations in HRV indices during exercise, it was not enough to significantly influence the changes in cardiac autonomic modulation. Throughout the recovery period, the hydration exercise protocol induced significant changes in cardiac autonomic modulation, promoting faster recovery of HRV indices, analyzed in the time and frequency domain.

## Competing interests

The authors of this manuscript declare that they have no competing interests.

## Authors’ contributions

All authors have made substantive intellectual contributions towards conducting the study and preparing the manuscript for publication. Specifically, IM participated in subject recruitment, acquisition of the data, preparing tables and figures for publication, interpretation of the data and all aspects of writing the manuscript. CP and LV were involved in concept and design of the study, gaining ethical clearance, interpretation of the data and all aspects of writing the manuscript; CF, VV and LA were co-authors, responsible for translate the manuscript to English and the revision of final manuscript. All authors read and approved the final manuscript.

## References

[B1] Maughan RJ Shirreffs SM Rehydration and recovery after exercise Sci Sport 2004 19 234 238 10.1016/j.scispo.2004.05.003 11131686

[B2] Sawka MN Montain SJ Latzka WA Hydration effects on thermoregulation and performance in the heat Comp Biochem Physiol A Mol Integr Physiol 2001 128 679 690 10.1016/S1095-6433(01)00274-4 11282312

[B3] Casa DJ Clarkson PM Roberts WO American College of Sports Medicine roundtable on hydration and physical activity: consensus statements Curr Sports Med Rep 2005 4 115 112 15907263 10.1097/01.csmr.0000306194.67241.76

[B4] Armstrong LE Maresh CM Gabaree CV Hoffman JR Kavouras SA Kenefick RW Castellani JW Ahlquist LE Thermal and circulatory responses during exercise: effects of hypohydration, dehydration, and water intake J Appl Physiol 1997 82 2028 2035 9173973 10.1152/jappl.1997.82.6.2028

[B5] Carter R III Cheuvront SN Wray DW Kolka MA Stephenson LA Sawka MN The influence of hydration status on heart rate variability after exercise heat stress J Thermal Biol 2005 30 495 502 10.1016/j.jtherbio.2005.05.006

[B6] Brouns F Nieuwenhoven MV Jeukendrup A Marken Lichtenbelt WV Functional foods and food supplements for athletes: from myths to benefit claims substantiation through the study of selected biomarkers Br J Nutr 2002 88 177 188 10.1079/BJN2002683 12495460

[B7] Coyle EF Fluid and fuel intake during exercise J Sports Sci 2004 22 39 55 10.1080/0264041031000140545 14971432

[B8] Jouven X Schwartz PJ Escolano S Straczek C Tafflet M Desnos M Empana JP Ducimetière P Excessive heart rate increase during mild mental stress in preparation for exercise predicts sudden death in the general population Eur Heart J 2009 30 1703 1710 10.1093/eurheartj/ehp160 19401600

[B9] Huikuri HV Castellanos A Myerburg RJ Sudden death due to cardiac arrhythmias N Engl J Med 2001 345 1473 1482 10.1056/NEJMra000650 11794197

[B10] Charkoudian N Halliwill JR Morgan BJ Eisenach JH Joyner MJ Influences of hydration on postexercise cardiovascular control in humans J Physiol 2003 552 635 644 10.1113/jphysiol.2003.048629 14561843 PMC2343381

[B11] Pardini R Matsudo SMM Matsudo VKR Araujo T Andrade E Braggion G Validation of the International Physical Activity Questionaire (IPAQ-version 6): pilot study in Brazilian young adults Rev Bras Ciên e Mov 2001 9 45 51

[B12] Tebexreni AS Lima EV Tambeiro VL Neto TLB Standard protocols in ergometry, practice implications versus ramp Rev Soc Cardiol Estado de São Paulo 2001 11 519 528

[B13] Vianna LC Oliveira RB Silva BM Ricardo DR Araújo CG Water intake accelerates post-exercise cardiac vagal reactivation in humans Eur J Appl Physiol 2008 102 283 288 17929050 10.1007/s00421-007-0584-7

[B14] Costill DL Sparks KE Rapid fluid replacement following thermal dehydration J Appl Physiol 1973 34 299 303 4688117 10.1152/jappl.1973.34.3.299

[B15] Von Duvillard SP Braun WA Markofski M Beneke R Leithäuser R Fluids and hydration in prolonged endurance performance Nutrition 2004 20 651 656 10.1016/j.nut.2004.04.011 15212747

[B16] Hernandez AJ Nahas RM Dietary changes, water replacement, food supplements and drugs: evidence of ergogenic action and potential health risks Rev Bras Med Esporte 2009 15 3 12

[B17] Armstrong LE Hydration assessment techniques Nutr Rev 2005 63 S40 54 16028571 10.1111/j.1753-4887.2005.tb00153.x

[B18] Task Force of the European Society of Cardiology of the North American Society of pacing electrophysiology Heart rate variability standards of measurement, physiological interpretation and clinical use Circulation 1996 93 1043 1065 10.1161/01.CIR.93.5.1043 8598068

[B19] Godoy MF Takakura IT Correa PR The relevance of nonlinear dynamic analysis (Chaos Theory) to predict morbidity and mortality in patients undergoing surgical myocardial revascularization Arquivos de Ciências da Saúde 2005 12 167 171

[B20] Corrêa PR Catai AM Takakura IT Machado MN Godoy MF Heart Rate Variability and Pulmonary Infections after Myocardial Revascularization Arq Bras Cardiol 2010 95 448 456 10.1590/S0066-782X2010005000123 20835682

[B21] Tarvainen MP, Niskanen JA, Lipponen PO, Ranta-aho & Karjalainen PA Kubios HRV – A software for advanced heart rate variability analysis 2008 Berlin: Springer: In: 4th European Conference os the International Federation for Medical and Biological Engineering, Sloten JV, Verdonck P, Nyssen M, Haueisen J, editors 1022 1025

[B22] Vanderlei LCM Pastre CM Hoshi RA Carvalho TD Godoy MF Basic notions of heart rate variability and its clinical applicability Rev Bras Cir Cardiovasc 2009 24 205 217 10.1590/S0102-76382009000200018 19768301

[B23] González-Alonso J Mora-Rodríguez R Below PR Coyle EF Dehydration markedly impairs cardiovascular function in hyperthermic endurance athletes during exercise J Appl Physiol 1997 82 1229 1236 9104860 10.1152/jappl.1997.82.4.1229

[B24] Crandall CG Zhang R Levine BD Effects of whole body heating on dynamic baroreflex regulation of heart rate in humans Am J Physiol Heart Circ Physiol 2000 279 H2486 2492 11045986 10.1152/ajpheart.2000.279.5.H2486

[B25] Boettger S Puta C Yeragani VK Donath L Müller HJ Gabriel HH Bär KJ Heart rate variability, QT variability, and electrodermal activity during exercise Med Sci Sports Exerc 2010 42 443 448 19952826 10.1249/MSS.0b013e3181b64db1

[B26] Perini R Veicsteinas A Heart rate variability and autonomic activity at rest and during exercise in various physiological conditions Eur J Appl Physiol 2003 90 317 325 10.1007/s00421-003-0953-9 13680241

[B27] Alonso DO Forjaz CLM Rezende LO Braga AM Barretto AC Negrão CE Rondon MU Heart rate response and its variability during different phases of maximal graded exercise Arq Bras Cardiol 1998 71 787 792 10347924 10.1590/s0066-782x1998001200008

[B28] Mendonca GV Fernhall B Heffernan KS Pereira FD Spectral methods of heart rate variability analysis during dynamic exercise Clin Auton Res 2009 19 237 245 10.1007/s10286-009-0018-1 19479301

[B29] Javorka M Zila I Balhárek T Javorka K Heart rate recovery after exercise: relations to heart rate variability and complexity Braz J Med Biol Res 2002 35 991 1000 10.1590/S0100-879X2002000800018 12185393

[B30] Sandercock GRH Brodie DA The use of heart rate variability measures to assess autonomic control during exercise Scand J Med Sci Sports 2006 16 302 313 10.1111/j.1600-0838.2006.00556.x 16774653

[B31] Casties JF Mottet D Le Gallais D Non-linear analyses of heart rate variability during heavy exercise and recovery in cyclists Int J Sports Med 2006 27 780 785 10.1055/s-2005-872968 16586334

[B32] Hamilton MT González-Alonso J Montain SJ Coyle EF Fluid replacement and glucose infusion during exercise prevent cardiovascular drift J Appl Physiol 1991 71 3 871 877 1757323 10.1152/jappl.1991.71.3.871

[B33] Rowland T Pober D Garrison A Cardiovascular drift in euhydrated prepubertal boys Appl Physiol Nutr Metab 2008 33 4 690 695 10.1139/H08-031 18641711

[B34] Coyle EF González-Alonso J Cardiovascular drift during prolonged exercise: new perspectives Exerc Sports Sci Rev 2001 29 2 88 92 10.1097/00003677-200104000-00009 11337829

[B35] Charkoudian N Eisenach JH Joyner MJ Roberts SK Wick DE Interactions of plasma osmolality with arterial and central venous pressures in control of sympathetic activity and heart rate in humans Am J Physiol Heart Circ Physiol 2005 289 H2456 2460 10.1152/ajpheart.00601.2005 16199481

[B36] Wenner MM Rose WC Delaney EP Stillabower ME Farquhar WB Influence of plasma osmolality on baroreflex control of sympathetic activity Am J Physiol Heart Circ Physiol 2007 293 H2313 2319 10.1152/ajpheart.01383.2006 17644564

[B37] Scrogin KE Grygielko ET Brooks VL Osmolality-: a physiological long-term regulator of lumbar sympathetic nerve activity and arterial pressure Am J Physiol 1999 276 R1579 1586 10362734 10.1152/ajpregu.1999.276.6.R1579

[B38] Yun AJ Lee PY Bazar KA Clinical benefits of hydration and volume expansion in a wide range of illnesses may be attributable to reduction of sympatho-vagal ratio Med Hypotheses 2005 64 646 650 10.1016/j.mehy.2004.07.014 15617881

[B39] Mountain SJ Cheuvront SN Sawka MN Exercise associated hyponatraemia: quantitative analysis to understand the aetiology Br J Sports Med 2006 40 2 98 105 10.1136/bjsm.2005.018481 16431994 PMC2492017

